# Ocean Acidification and the Loss of Phenolic Substances in Marine Plants

**DOI:** 10.1371/journal.pone.0035107

**Published:** 2012-04-25

**Authors:** Thomas Arnold, Christopher Mealey, Hannah Leahey, A. Whitman Miller, Jason M. Hall-Spencer, Marco Milazzo, Kelly Maers

**Affiliations:** 1 Department of Biological Sciences, Dickinson College, Carlisle, Pennsylvania, United States of America; 2 Smithsonian Environmental Research Center, Edgewater, Maryland, United States of America; 3 School of Marine Science and Engineering, University of Plymouth, Plymouth, United Kingdom; 4 Dipartimento di Scienze della Terra e del Mare, University of Palermo, Palermo, Italy; Swansea University, United Kingdom

## Abstract

Rising atmospheric CO_2_ often triggers the production of plant phenolics, including many that serve as herbivore deterrents, digestion reducers, antimicrobials, or ultraviolet sunscreens. Such responses are predicted by popular models of plant defense, especially resource availability models which link carbon availability to phenolic biosynthesis. CO_2_ availability is also increasing in the oceans, where anthropogenic emissions cause ocean acidification, decreasing seawater pH and shifting the carbonate system towards further CO_2_ enrichment. Such conditions tend to increase seagrass productivity but may also increase rates of grazing on these marine plants. Here we show that high CO_2_ / low pH conditions of OA *decrease,* rather than increase, concentrations of phenolic protective substances in seagrasses and eurysaline marine plants. We observed a loss of simple and polymeric phenolics in the seagrass *Cymodocea nodosa* near a volcanic CO_2_ vent on the Island of Vulcano, Italy, where pH values decreased from 8.1 to 7.3 and pCO_2_ concentrations increased ten-fold. We observed similar responses in two estuarine species, *Ruppia maritima* and *Potamogeton perfoliatus*, in *in situ* Free-Ocean-Carbon-Enrichment experiments conducted in tributaries of the Chesapeake Bay, USA. These responses are strikingly different than those exhibited by terrestrial plants. The loss of phenolic substances may explain the higher-than-usual rates of grazing observed near undersea CO_2_ vents and suggests that ocean acidification may alter coastal carbon fluxes by affecting rates of decomposition, grazing, and disease. Our observations temper recent predictions that seagrasses would necessarily be “winners” in a high CO_2_ world.

## Introduction

Increasing levels of atmospheric CO_2_ can trigger accumulations of plant phenolic substances such as lignins, tannins, and phenolic acids and glycosides which serve as structural or chemical defenses against grazers and disease organisms [1]–[6]. In a meta-analysis of over one hundred separate studies Stiling and Cornelissen found that elevated CO_2_ tends to increase plant C/N ratios and trigger the accumulation of tannins and other phenolics while also having significant effects on the abundance, consumption rates, development times, relative growth rates, conversion efficiencies, and pupal weights of a broad range of herbivores [7]. CO_2_ enrichment can also alter the characteristics of leaf litter, inhibiting the activity of detritivores [1], [8]. CO_2_-stimulated accumulations of plant phenolics are often predicted by popular models of plant defense, especially resource availability models linking excess CO_2_ and carbohydrates to an increased production of carbon-based defenses [9], [10].

The availability of CO_2_ is also increasing dramatically in oceans and estuaries. About a third of anthropogenic carbon emissions have been absorbed by the oceans, driving the process of ocean acidification wherein absorbed CO_2_ generates carbonic acid, increasing the concentrations of H^+^, HCO_3_^−^, and dissolved CO_2_, while lowering CO_3_^2−^ concentrations and seawater pH [11]. In 150 years, the average ocean pH has dropped from 8.21 to 8.10 [12]. By the end of this century seawater pH is expected to fall another 0.3 to 0.4 units, leading to a 150% increase in H^+^ and a corresponding increase in available CO_2_ of ∼300–400%. Acidification also occurs in estuaries where trends of decreasing pH have been detected amid the daily fluctuations driven by biological processes and tides [13], [14]. Such high CO_2_ / low pH conditions can stimulate the productivity of many marine photoautotrophs, including seagrasses which lack effective carbon-concentrating mechanisms [15]–[20]. For example, in mesocosm experiments CO_2_ enrichment resulted in increases in photosynthesis, reproductive output, and carbohydrate levels in eelgrass, *Zostera marina* [21]. Similarly, studies of high CO_2_ communities near submerged volcanic vents reveal luxurious seagrass beds with increased shoot densities and biomass, and leaves devoid of calcifying fouling organisms [22]–[24].

We have observed that seagrasses growing near undersea volcanic vents exhibit a greater-than-usual number of grazing scars and wondered if high CO_2_ / low pH conditions may affect the value of seagrass as a food item for herbivores. Such conditions may enhance the value of seagrasses as a food item by reducing the presence of calcareous epiphytes [22], altering tissue nutrients contents, or affecting the production of chemical and structural deterrents. The primary deterrent substances in seagrasses and most estuarine plants are phenolics, including simple and conjugated phenolic acids, condensed tannins, and lignins implicated as herbivore deterrents, digestion reducers, and antifoulants [25]–[34]. Many phenolics have antimicrobial properties; for example, phenolic acids from seagrasses inhibit the growth of the marine pathogen *Labyrinthula* which causes the seagrass wasting disease [35]–[37]. These compounds are synthesized via the shikimic acid and phenylpropenoid (SA/PP) pathway which is up-regulated by CO_2_, visible and UV light, and photosynthesis, and sugars [27], [38]. This suggests that the high CO_2_ / low pH conditions of ocean acidification may trigger accumulations of these carbon-based chemical defenses in marine vascular plants, a prediction that is seemingly inconsistent with our previous observations of increased grazing near volcanic vents.

To test the assumption that ocean acidification would trigger accumulations of phenolic substances we examined seagrasses inhabiting the CO_2_-enriched waters near an underwater volcanic seep on the Island of Vulcano, Italy and submerged aquatic vegetation in tributaries of the Chesapeake Bay, USA where ocean acidification was simulated using Free-Ocean-Carbon-Enrichment (F.O.C.E.).

## Methods

### Natural CO_2_ vent site

**Figure 1:**
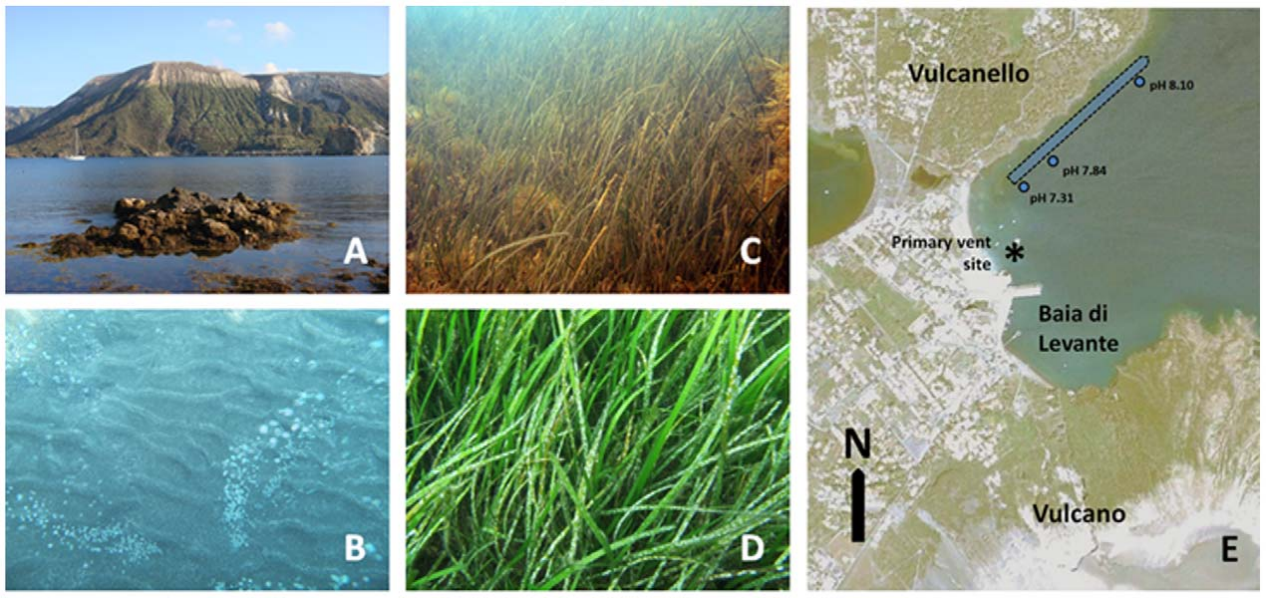
Location of the underwater CO_2_ vent and seagrass meadows on the Island of Vulcano, Italy. (A) Location of near shore transect beneath the volcano, where dense populations of *Cymodocea nodosa* inhabit shallow grass beds (C,D). (E) The sampling sites spanned a range of high CO_2_/low pH conditions at distances of 260, 300 and 380 m from the vent site (*) along the northern shoreline of the bay.

Several submersed CO_2_ vent systems are present in the Aeolian archipelago (Northeastern Sicily, Italy), generated by subduction processes in the Southern Tyrrhenian seafloor [39], [40]. The southernmost volcanic island of Vulcano (38°25′08.52”N, 14°57′39.13”E) contains the most recently active center in the Gran Cratere at the top of the Fossa cone (last eruption 1888–1890) and several minor volcanic centers [41]. Most of the intense submersed seeps are located along southern and western shores of Baia di Levante (38°25′01.44”N, 14°57′36.29”E), where dispersed underwater leaks cover a 130×35 m shallow area (<1m depth) (Figure 1).

The Vulcano CO_2_-seeps are particularly well-suited for studies of future ocean acidification. Gas composition at the seeps consists of >99% of carbon dioxide [42]. Dissolved hydrogen sulphide from the seeps, potentially toxic for cellular respiration, does not extend to the study sites. For example, while H_2_S was found at a concentration of 273 and 166 µmol/Kg inside an intense bubbling site [43] it was undetectable (<2 ppm) at the sampling locations >20 m away (Parello et al. in prep.). At these distances sulphate (SO_4_^–^) levels are typical for oceanic waters (Parello et al. in prep.). This suggests that only a small proportion of the H_2_S enters into the aquatic phase, where it oxidizes to non-toxic sulfate due to the high O_2_ saturation (∼100%) recorded across the bay.

A CO_2_/pH gradient runs parallel to the northwestern coast of the Baia di Levante. The pH at the emission site ranges from 5.2 to 5.5 and the gradient reaches an ambient pH (∼8.1 pH) at >350 m from an intense CO_2_ leakage site. This CO_2_ gradient encompasses a great variety of intertidal and subtidal communities at <2 m depth (Figure 1). *Cymodocea nodosa* occurs along the CO_2_ gradient and has long been the dominant seagrass in the bay [44].

In May 2011 we collected leaf tissues from *C. nodosa* at 380, 300, and 260 m distances from the seep. Here, average pH values decrease from 8.1 to 7.3 and *p*CO_2_ concentrations increased ten-fold from 422 to 4009 ppm, spanning both present-day conditions as well as those predicted for the next 150 years.

### Free ocean carbon enrichment

**Figure 2:**
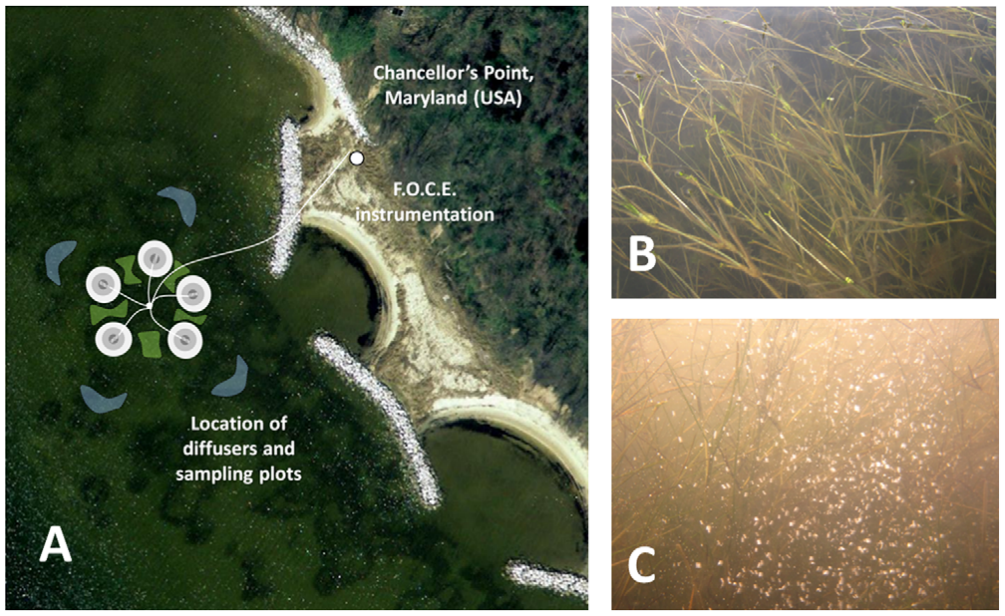
Location of mid-salinity Free Ocean Carbon Enrichment (FOCE) experiments within the St. Mary's River, Maryland (USA) in May-July 2010. (A) Individual CO_2_ diffusers (white) generated halos of high CO_2_/low pH conditions (5 cm and 40 cm distances in grey; 100 cm locations in green; 500 cm ambient sites in blue.) (B) Dense meadow of *Ruppia maritima* from the near shore site. (C) Bubbles emitted from one of the CO_2_ diffusers (compare to CO_2_ natural vent site, Fig 1.B).

**Figure 3:**
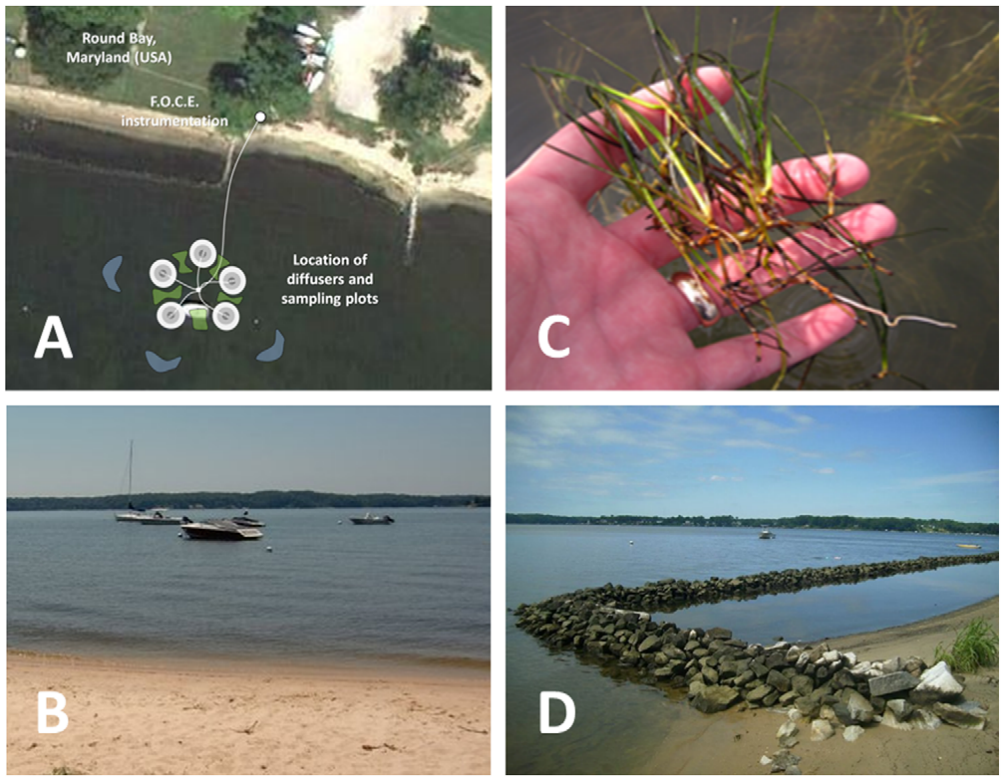
Location of low-salinity Free Ocean Carbon Enrichment (FOCE) experiments in the Severn River, Maryland (USA) in June-July 2011. (A) Individual CO_2_ diffusers (white) generated halos of high CO_2_/low pH conditions (5 cm and 40 cm distances in grey; 100 cm locations in green; 500 cm ambient sites in blue.) The FOCE system instrument package was located on shore (B,D) and maintained high CO_2_/low pH conditions within mixed-species meadows of widgeon grass *(Ruppia maritima)* and redhead grass *(Potamogeton perfoliatus)* (C).

Long-term manipulative experiments were conducted in tributaries of the Chesapeake Bay, USA, including the St. Mary's River (38°10'1.44"N, 76°26'33.50"W) in 2010 and the Severn River (39° 3'31.73"N, 76°32'38.17"W) in 2011. These experiments were conducted during the growing season (May-July) of each year. The St. Mary's River site contains perennial beds of Widgeon Grass *Ruppia maritima* (long form) at a depth of 1–2 m and salinities of 15–25 ppt (Figure 2). Here plants were acclimated to high CO_2_/low pH conditions for four weeks prior to harvesting. The Severn River site includes a mixed grass bed of Widgeon Grass, *Ruppia maritima* (short form), and Redhead Grass, *Potamogeton perfoliatus* at similar depths and salinities of 4–7 ppt (Figure 3). At this site, high CO_2_/low pH conditions were maintained for 1.5 mo with sampling occurring twice, at 4 and 6 wks.

The Free-Ocean-Carbon-Enrichment (F.O.C.E.) system was designed for *in situ* experiments requiring the manipulation of ocean pH under otherwise natural conditions. The process is analogous to free-air-carbon-enrichment (F.A.C.E.) commonly used to test the effects of atmospheric CO_2_ increases on land. This portable F.O.C.E. system can be configured in several different ways to deliver either CO_2_ gas or CO_2_-enriched seawater on demand. Here, the system was configured to supply compressed CO_2_ to five underwater diffusers, as determined by a computer-controlled 2.5 W solenoid. The electrical system can be located on site within a custom support buoy or on shore. Power is supplied by an internal 12V DC 78 amp-hour sealed AGM deep cycle battery mated to a Morning Star pure sine wave DC-to-AC inverter. Batteries can be charged by a 125 W Kyocera solar array with a 12V MPPT charge controller (Morning Star Corp., USA). For safety, the electronics and batteries are secured in a waterproof but vented enclosure; compressed gas cylinders must be protected from temperature extremes and gases produced by battery recharging must be exhausted. During these experiments, CO_2_ flowed at 19 psi on a 20s/20s on/off cycle through 65–90 m of gas line to an underwater gang valve junction, and then to one of five replicate CO_2_ diffusers secured within a 14×14 m experimental plot. Diffusers were anchored at similar depths and were separated by ≥5 m. Release of pure CO_2_ creates a gentle stream of bubbles, similar to those found at the natural CO_2_ vent sites. The system is capable of reducing seawater pH by >4.5 units; however, for these experiments the system was programmed to maintain a drop of ∼0.5 pH units, roughly doubling pCO_2_ levels, at a distance of 40 cm from each injector. For example, monitoring of multiple diffuser sites in July 2011 indicated average reductions in pH of 0.61 units with pCO_2_ values 3.2 times ambient levels at these distances. In 2010, an average reduction of 0.41 pH units and a 3.0-fold increase in pCO_2_ was recorded. This mimics the change in seawater chemistry and drop in surface ocean pH predicted for the next century under a business-as-usual scenario and span the range of pH used in previous mesocosm studies. Conditions were monitored at distances of 5, 40, 100, and 500 cm from each CO_2_-injector, where plants were later harvested. At these distances we noted variations in carbonate chemistry that are the norm for estuarine systems, including those associated with tides, diurnal cycles, and a putative brown tide event on July 11, 2011 (note higher than typical ambient pH). The F.O.C.E system maintained a pH drop of ∼0.5 units relative to this natural variation, day and night. We also verified that dissolved oxygen concentrations were not affected by the F.O.C.E. system. The carbonate system at these sites was determined by analyses of discrete water samples, real-time analyses of pCO_2_ concentrations using an underway flow-through system, a solid-state pH probe, and – for comparison – a traditional glass electrode probe. Parameters not directly measured were calculated using the CO2SYS1.01 program (http://cdiac.ornl.gov.ftp.co2sys/.).

**Table 1:** Concentrations of phenolic substances in *Cymodocea nodosa* shoots collected at various distances from the natural CO_2_ vent site from the Island of Vulcano, Italy in May 2011.

Conditions	Seawater carbonate chemistry		
Distance from seep	380m	300m	260m
Salinity	37.16±0.07	37.12±0.06	37.05±0.1
pH (units)	8.11±0.01	7.84±0.04	7.32±0.05
pCO_2_ (µtm)	422±43	976±269.5	4009±1442.7
TA (µmol kg^-1^)	2549.6±29.6	2555.9±28.9	2592.5±48.3

Cymodocea nodosa	Product concentration (mg g^−1^ WM)			General trend	Test	P
Proanthocyanindins	13.62±1.66	10.17±0.73	10.92±0.87	25% decrease*	**1**	**0.088**
Total phenolic acids	4.66±0.59^ a^	5.30±1.45^ ab^	1.89±0.19^ b^	59% decrease**	**2**	**0.017**
Gallic acid	0.50±0.05	0.41±0.03	0.50±0.04	*no change*	1	0.252
Syr+4-HBA	0.07±0.01^ a^	0.05±0.01^ ab^	0.03±0.01^ b^	58% decrease**	**1**	**0.006**
Vanillin	1.02±0.65	2.57±1.38	0.36±0.24	*65% decrease*	2	0.344
Acetovillinone	3.06±0.88	2.27±1.47	1.00±0.25	*67% decrease*	1	0.413
Coumaric acid	0.29±0.16	0.16±0.05	0.17±0.09	*41% decrease*	2	0.882
Ferulic acid	0.38±0.18	0.31±0.11	0.19±0.09	*50% decrease*	2	0.195
All phenolics†	109.30±4.39^ a^	104.51±4.48^ ab^	93.12±4.26^ b^	15% decrease**	**3**	**0.026**

Values are means +/− SE.

Average (±SE) temperature, salinity and pH were collected on different visits from Sept 2009 to May 2011 (n = 60). Total Alkalinity (TA) was calculated from water samples collected at each site on April and November 2010 (n = 4). Statistical analyses: 1, one-factor ANOVA with Holm-Sidak multiple comparisons; 2, Kruskal-Wallis One Way Analysis of Variance on Ranks with Tukey or Dunns multiple comparisons; 3, Two-factor ANOVA on ranks with Holm-Sidak multiple comparisons. Letters indicate results of pairwise comparisons test *P*<0.05. ^†^a combined analysis indicated significant variation in the overall concentrations of the various compounds (factor: compound type; *P*<0.001) but there was no significant difference in how the compound types responded to changes in pH (interaction term; *P* = 0.900).

### Permits

All necessary permits were obtained for the described field studies.

The harvesting of plant samples from the Chesapeake Bay sites were permitted by the Maryland Department of Natural Resources, and coordinated with local landowners including St. Mary's College of Maryland and Historical St. Mary's City the Round Bay Community Association in Round Bay, Maryland.

### Seawater carbonate analyses

At each field site the seawater carbonate system was characterized multiple times, using a range of analytical methods. At the Baia di Levante location (Island of Vulcano, Italy), a 556 MPS YSI (Yellow Springs, USA) probe was used for rapid assessment of pH, salinity (ppt) and temperature (°C). Continuous measures were also collected in November 2010 and May 2011 at 15-min intervals for the at least 24 h. In common with other recent volcanic vents studies [23], [45], [46] we recorded pH fluctuations along a shallow CO_2_ gradient in different weather conditions and at various hours of the day. On a regular basis (approximately every 45 days) from September 2009 to May 2011, the various seawater parameters were recorded in triplicates at 380 m (n = 60), 300 m (n = 60), and 260 m (n = 63) sites. The pH meter was accurate to 0.01 pH units and previously calibrated using TRIS/HCl and 2-aminopyridine/HCl buffer solutions [47]. For each site, pH means were calculated from hydrogen ion concentrations before re-converting back to pH values [45]. Water samples for Total Alkalinity (TA) were collected at each site along the pH gradient on April and November 2010 (n = 4) from a 100 ml water sample passed through 0.2 µm pore size filters, poisoned with 0.05 ml of 50% HgCl_2_ to avoid biological alteration, and then stored in the dark at 4°C. Three replicate sub-samples were analyzed at 25°C using a titration system. The pH was measured at 0.02 ml increments of 0.1 N HCl. Total alkalinity was calculated from the Gran function applied to pH variations from 4.2 to 3.0, as mEq Kg^−1^ from the slope of the curve HCl volume versus pH. At the Chesapeake Bay sites the carbonate system at various distances from the diffusers was characterized periodically before and during the experiments, using several complimentary methods. During installation, pH was measured over periods of seconds to days using a YSI 556 MPS probe and a solid state probe (Honeywell Durafet II). The pH probes were calibrated with NIST traceable buffers (pH = 7.00 and 10.00) and measurements were made on the NBS scale). During experiments, more complete measurements were conducted. A portable flow-through underway pCO_2_ system was used to measure real time pCO_2_ values *in situ*, at the prescribed distances from CO_2_ diffusers (in all four compass headings) and at representative control sites. The instrument design was based on the Palmer underway pCO_2_ system produced by the Lamont-Doherty Earth Observatory with some modification to enable the system to be easily deployed in a small boat. The seawater equilibrator was based on the design for rapid equilibration under relatively low flow by W. McGillis (J. Salisbury, pers. comm.) and the infra-red gas analyzer was a dual (NDIR) model (CO_2_meter.com) which logs CO_2_ concentration, relative humidity, and temperature at 2-min intervals to a custom microprocessor designed specifically for this instrument. Total alkalinity (TA) samples were taken and processed via the spectrophotometric method of Yao and Byrne using an Ocean Optics (model USB2000) spectrophotometer [48]. TA and pCO_2_ were used as master variables in CO2sys.xls to characterize the carbonate chemistry of the system. Dissociation constants K_1_ and K_2_ for carbonic acid in estuarine waters of Cai and Wang and NBS pH scale were selected to run the CO2sys model [49]. Here we present representative data for the St. Mary's River site collected at a range of distances from a single CO_2_ diffuser in June 2010. For the Severn River site we present representative data collected from similar distances from multiple CO_2_ diffusers in June 2010.

### Biochemical analyses

Biochemical analyses of natural products were conducted for replicate plant tissues harvested from each site. Individual leaves (*C. nodosa*) or shoots with roots/rhizomes (*R. maritima, P. perfoliatus*) were harvested from each location, wiped clean of epiphytes, and analyzed separately. For *C. nodosa* the 2^nd^ rank leaves were collected from separate shoots and identical 7 cm midsections were dissected. Leaf sections from three different shoots were pooled for each extraction. Separate extractions were analyzed for condensed tannins (n = 16 to 18/location) and phenolic acids (n = 8/location). For samples from the Chesapeake Bay, whole plants were collected from the specified distances from each CO_2_ diffuser, and from control areas. At the St. Mary's River site 405 shoots were harvested at three distances from the five CO_2_ diffusers, for a total of 15 locations. From each location, tissues from 27 shoots were pooled, 3 per extraction, to generate 9 replicate extractions. At the Severn River site plants were harvested at either three or four distances from the five CO_2_ diffusers, for a total of 15–20 sampling locations sampled at each time interval. This resulted in the sampling of >900 *R. maritima* shoots in total. Fewer *P. perfoliatus* samples were obtained, as this species did not occur at every location. These tissues were pooled for extraction as described above, providing 9–15 extractions per location at each sampling interval. All samples were transported at −80°C, homogenized, and extracted in MeOH(aq) with 2% acetic acid for 24 h at 4°C in the dark. Concentrations of phenolic acids were determined by RP-HPLC using a method modified from previous studies [50], [51]. One hundred μl of each extract was filtered to remove particulate matter and injected onto a semi-preparative RP-18 HPLC column (Supleco, Belfonte PA). We modified our previous methods to employ a gradient system, which more effectively resolved phenolic acid peaks from these species. Peaks were identified by comparison to commercial standards and concentrations (mg compound g^−1^ blade DM) determined using individual standard curves. Condensed tannins (proanthocyanindins), were quantified using a micro-titer plate assay derived from the acid-butanol method [52], [53]. Total reactive phenolics were determined using a micro-Folin-Denis assay [54]. Standard curves were developed using quebrancho tannin obtained from A. Hagerman (Miami University of Ohio). Natural product concentrations were expressed as mg compound g^−1^ tissue wet mass. Our selection of these natural products for the analyses was based primarily upon their concentrations and previous reports of their putative roles as antimicrobials or antifeedants; however, we also sought to quantify concentrations of related compounds that are important precursors for phenolic biosynthesis, whether or not they have demonstrated bioactivity themselves.

### Statistical analyses

Statistical analyses were conducted using SigmaStat. In experiments examining three sampling sites groups were compared using ANOVAs with Holm-Sidak multiple comparisons or, when transforming data did not satisfy test assumptions, with Kruskal-Wallis One Way Analysis of Variance on Ranks with Tukey or Dunns multiple comparisons. For experiments comparing only two sites datasets were compared using Student's t-test or a Mann-Whitney Rank Sum Test. An α level of 0.05 was used to determine significance. P values between 0.05 and 0.10 are also noted in the tables.

## Results

In these natural and manipulative experiments, we observed that high CO_2_/low pH conditions were associated with a dramatic loss, rather than the predicted accumulations, of tannins and related phenolics in undersea vegetation. In experiments conducted at three sites, including high salinity and estuarine areas, we detected reduced levels of proanthocyanidins, hydrozybenzoates, hydroxycinnamates, and total reactive phenolics in three species of aquatic vascular plants.

**Table 2:** Concentrations of phenolic substances in the “long” form of widgeon grass subjected to Free Ocean Carbon Enrichment within the St. Mary's River, Maryland (USA) in May-July 2010.

Conditions	Seawater carbonate chemistry		
Distance from injector	500cm	40cm	5cm
Temperature (C)	25.0	25.0	25.0
Salinity	17	17	17
pH	8.4	8.0	6.9
pCO_2_ (µtm)	157.8	469.3	6792.0
TA (µmol kg^−1^)	1467.0	1444.0	1455.0

*Ruppia maritima* (long)	Product concentration (mg g^−1^ WM)			General trend	Test	*P*
Proanthocyanindins	5.92±0.99^a^	4.69±.068^ a^	2.41±0.31^ b^	60% decrease**	**2**	**0.002**
Total reactive phenolics	3.43±.30^ a^	1.67±.26^ b^	1.88±.31^ b^	45% decrease**	**1**	**<0.001**
Coumaric acid	0.31±.05^ a^	0.18±.01^ ab^	0.15±0.01^ b^	53% decrease**	**2**	**<0.001**

Values are means +/− SE.

Statistical analyses: 1, one-factor ANOVA with Holm-Sidak multiple comparisons; 2, Kruskal-Wallis One Way Analysis of Variance on Ranks with Tukey or Dunns multiple comparisons. Letters indicate results of pairwise comparisons test *P*<0.05.

**Table 3:** Concentrations of phenolic substances in low-salinity populations within the Severn River, Maryland (USA) after four weeks of Free Ocean Carbon Enrichment in June-July 2011.

Conditions	Seawater carbonate chemistry			
Distance from injector	500cm	100cm	40cm	5cm
Temperature (C)	28.3	28.3	28.3	28.3
Salinity	4.3	4.3	4.3	4.3
pH	8.34±0.01	8.26±0.02	7.82±0.04	7.32±0.06
pCO_2_ (µatm)	243±9	295±13	948±89	3465±527
TA (µmol kg^−1^)	1122±0.5	1122±0.5	1122±0.5	1122±0.5

*Ruppia maritima* (short)	Tissue	Product concentration (mg g^−1^ WM)		Trend	Test	*P*
		100cm	40cm			
Proanthocyanindins	whole plants	25.00±7.19	6.33±0.55	75% decrease**	**1**	**0.018**
Syr+4-HBA	shoots	0.08±0.03	0.27±0.10	330% increase*	1	**0.081**
	roots	0.07±0.01	0.08±0.02	*no change*	1	0.647
Vanillin	shoots	0.02±0.02	0.05±0.03	*no change*	2	0.415
	roots	ND	ND	*no change*	ND	ND
Acetovillinone	shoots	0.08±0.05	0.05±0.02	*no change*	2	0.917
	roots	0.13±0.10	0.18±0.05	*no change*	2	0.210
Coumaric acid	shoots	ND	<0.01	*no change*	2	0.424
	roots	<0.01	<0.01	*no change*	2	0.797
Ferulic acid	shoots	0.03±0.00	0.03±0.00	*no change*	1	0.451
	roots	0.01±0.00	0.02±0.00	*no change*	1	0.185

Values are means +/− SE.

ND, not detected. Statistical analyses: 1, Student's t-test; 2, Mann-Whitney Rank Sum Test; *P*<0.05.

In the Mediterranean, we found that levels of phenolic substances were significantly decreased in *Cymodocea nodosa* near CO_2_ vents on the island of Vulcano, Italy (Table 1). Along a 120 m underwater transect levels of pCO_2_ increased ten-fold from 422 to 4008 μatm, with a corresponding reduction of 0.8 pH units. Over this distance concentrations of proanthocyaninidins and total phenolic acids decreased by 25% and 59%, respectively. We also detected decreased levels of specific hydroxycinnamic acid– and hydroxybenzoic acid-derivatives, including syringaldehyde and 4-hyroxybenzoic acid. Some related compounds, e.g. gallic acid, vanillin, acetovinillone, and coumaric acid, were unaffected but, importantly, no compound or class of compounds increased in concentration under high CO_2_/low pH conditions.

At estuarine sites we observed similar responses for *Ruppia maritima* and *Potamogeton perfoliatus*, two important submerged plants in Chesapeake Bay. In the polyhaline reach of the St. Mary's River (Maryland, USA) we detected a dramatic loss of phenolic substances in the “long form” of *Ruppia* after 63 days of CO_2_ enrichment (Table 2). Plants located 40 and 5 cm from the F.O.C.E. CO_2_-injectors (pH 8.0 and 6.9, respectively) possessed 45–60% lower levels of proanthocyanindins, *p*-coumaric acid, and total reactive phenolics than did control plants located 5 m from each injector (pH 8.4). We observed that daytime pCO_2_ levels in these dense meadows site were ≤200 µatm due to uptake of CO_2_ by rapid photosynthesis; the F.O.C.E. system successfully counteracted this, maintaining pCO_2_ levels 2–3 times higher than ambient at a distance of 40 cm. During this two-month period plants grew >1m in height and became reproductive, bearing iridescent green flowers and semi-transparent seed pods. We observed no differences in the prevalence of reproductive structures (bud, flowers, or seed pods; data not shown) for CO_2_ enriched plants compared to those outside the experimental areas. In the low salinity Severn River (Maryland, USA) we also detected CO_2_-induced decreases in phenolics in the “short” form of *R. maritima* and in *P. perfoliatus*. After only 18 days, levels of proanthocyanindins dropped 75% in the leaves and rhizomes of *R. maritima* located 40 cm from CO_2_ injectors, compared to those 150 cm away (Table 3). Phenolic acids in leaves or rhizomes were unchanged, except for a marginally significant increase in one phenolic acid present at trace amounts (<0.03% plant wet mass). By day 28, dramatically reduced phenolic levels of 61–85% were detected in Redhead Grass, *P. perfoliatus*, which exhibited lower levels of proanthocyanindins and total reactive phenolics near the CO_2_ injectors (Table 4). Altogether, *R. maritima* and *P. perfoliatus* exhibited either a loss of phenolics at high CO_2_ / low pH sites or no change at all.

**Table 4:** Concentrations of phenolic substances in low-salinity populations within the Severn River, Maryland (USA) after six weeks of Free Ocean Carbon Enrichment in June-July 2011.

Conditions	Seawater carbonate chemistry		
Distance from injector	500cm	100cm	40cm
Temperature (C)	29.5	29.5	29.5
Salinity	4.9	4.9	4.9
pH	8.29±0.01	8.11±0.02	7.94±0.02
pCO_2_ (µtm)	279±2	439±20	729±5
TA (µmol kg^−1^)	1145±10	1145±10	1145±10

*Ruppia maritima* (short)	Tissue	Product concentration (mg g^−1^ WM)			Trend	Test	*P*
		100cm	40cm	5cm			
Proanthocyanindins	shoots	1.76±0.39	1.24±0.28	2.44±1.29	*no change*	4	0.519
	roots	17.29±2.95^ a^	22.81±1.46^ b^	18.12±1.68^ a^	intermediate**	**4**	**0.015**
Syr+4-HBA	shoots	ND	ND	ND	*no change*	ND	ND
Vanillin	shoots	ND	ND	ND	*no change*	ND	ND
Acetovillinone	shoots	0.07±0.02	0.07±0.02	0.07±0.02	*no change*	3	0.986
Coumaric acid	shoots	0.02±0.01	0.01±0.00	0.01±0.00	*no change*	3	0.960
Ferulic acid	shoots	0.02±0.00	0.02±0.00	0.02±0.00	*no change*	3	0.922

*Potamogeton perfoliatus*							
Proanthocyanindins	whole plants	0.65±0.44^ a^	ND^ b^	0.10±0.10^ b^	85% decrease**	**3**	**0.016**
Total reactive phenolics	whole plants	2.13±0.69	0.78±0.35	0.85±0.33	61% decrease*	**4**	**0.100**

Values are means +/− SE.

ND, not detected. Statistical analyses: 3, Kruskal-Wallis One Way Analysis of Variance on Ranks with Tukey or Dunns multiple comparisons; 4, One-factor ANOVA with Holm-Sidak multiple comparisons. Letters indicate results of multiple pairwise comparisons tests, *P*<0.05.

In four populations of undersea vegetation from three different locations we observed eleven instances of CO_2_-induced reductions in phenolics. This response is in stark contrast to the CO_2_-induced accumulations of phenolics typically observed in terrestrial plants and predicted by popular models of plant defense.

## Discussion

Our results demonstrate that ocean acidification can decrease levels of phenolic protective substances in marine and estuarine plants, the opposite effect to that typically observed for land plants exposed to atmospheric CO_2_ enrichment. Dramatic reductions occurred in all four of the seagrass populations we tested, including those acclimated to naturally acidified conditions near volcanic vents and those exposed to free ocean carbon enrichment.

The phenolic contents of seagrasses, together with nitrogen contents and toughness, determine their palatability and grazing by isopods, urchins, fish, waterfowl, and turtles [55]–[57]. For example, Verges et al. demonstrated that chemical defenses from the seagrass *Posidonia oceanica* dramatically reduced the feeding of a wide range of consumers, including fishes and sea urchins [55]. More recently, Tomas et al. found that herbivorous isopods preferred tissues of eelgrass, *Zostera marina*, with low phenolic contents, and correspondingly high nutritional values [58]. Leaf toughness, which is determined in part by phenolic polymers such as lignin, is also an important determinant of herbivore feeding, especially for omnivorous fish [56]. Phenolics are important for mesohaline species such as *Ruppia spp.* and *Potamogeton spp.* which are noted for their high tannin levels and specialized brown “tannin cells”. Den Hartog and Kuo classified these plants as “eurysaline” species rather than true seagrasses, noting that many can tolerate a broad range of salinities but are usually not able to compete successfully with the seagrasses under oceanic conditions [59]. *R. maritima* inhabits temperate, tropical, and polar regions in part because of an impressive ability to tolerate different salinities, from brackish waters to salt ponds with salinities three times higher than the open oceans. Many of these “eurysaline” species are grazed heavily by water birds and invertebrates, which consume shoots, rhizomes, and seeds [60]–[63]. For example, at some locations in the Baltic Sea the exclusion of waterfowl resulted in up to an 80-fold increase in the density of *Potamogeton* sp. [61]. These eurysaline plants produce a variety of bioactive natural products, including simple and polymeric phenolics, which affect the feeding, digestion, and growth rates of these grazers. For example, Dorenbosch and Bakker conducted feeding experiments with five species of submerged macrophytes, including *Potamogeton pectinatus*, and noted that high phenolic species were the least grazed by omnivorous rudd and herbivorous grass carp [64]. Elkin et al. found that dietary tannins reduced the growth of waterducks and developing chicks by as much as a third [65]. Phenolics are not effective against all grazers however; for example, Cronin and Lodge reported that differences in the phenolic contents of *Potamogeton amplifolius* did not influence grazing by a freshwater crayfish [66].

Seagrasses also produce numerous antimicrobial substances, some of which are phenolics [67]–[69]. For example, caffeic acid and related phenolics inhibit the growth of *Labyrinthula*, a slime-mold like pathogen that causes periodic mass die-offs of certain seagrasses such as *Zostera* and *Thalassia* [37]. Decreased phenolic levels have been linked with outbreaks of the wasting disease in *Zostera marina* [70]. It has also been proposed that this pathogen spreads more quickly when shoot density (and, thus, blade-to-blade contact) is high, as they are at many CO_2_-enriched sites [26]. Additional work is warranted to determine if high CO_2_/low pH conditions may affect the phenolic substances of other seagrass genera, such as *Zostera* or *Thalassia,* in a way that could promote large scale die-offs associated with the seagrass wasting disease.

In general, the roles of phenolics in seagrasses and aquatic plants are analogous to those of terrestrial plants, where they act as antimicrobials and as deterrents and digestion reducers for many, but not all, invertebrate and vertebrate grazers [71]–[73]. Their relative importance compared to other factors is difficult to determine, especially since plant phenolic concentrations, nitrogen contents, and toughness are interrelated. However, in plant-animal interactions where phenolics are influential, their bioactivity is dosage-dependent; as a result, the observed reductions may help to explain our observations of increased rates of fish grazing near CO_2_ vent sites. They may influence rates of herbivory and disease, important contributors to present-day seagrass declines [55], [74]–[75].

The metabolic mechanism by which CO_2_-enrichment caused dramatic reductions in seagrass phenolic contents remains unknown and deserves further study. The shikimic acid / phenylpropenoid pathway in plants leads to the deamination of the amino acid phenylalanine, providing the carbon skeletons required for phenolic biosynthesis [38], [72]. Phenylalanine is a common precursor required both for the synthesis of phenolics and the proteins necessary for plant growth; as a result, these processes compete for resources and are often inversely correlated [76]. The notion that plants allocate finite resources to competing primary and secondary processes has been at the center of plant defense theory for the past half a century [77]–[80]. Many of these theories predict that excess carbon beyond that which can be used for plant growth is redirected to secondary metabolic processes, which in turn protect plant tissues when they may be most difficult to replace. Indeed, high levels of CO_2_ and sugars as well as high irradiances – which result in elevated tissue carbon:nitogen ratios – often trigger accumulations of plant phenolics. For instance, Cronin and Lodge reported that for *Potamogeton amplifolius* C:N ratios were increased 55% and leaf phenolics were increased 72% by high light [66]. However, when growth is not nutrient-limited these models instead predict the allocation of carbon to protein synthesis and growth [76]. In fact, in well-fertilized and rapidly growing plants, including seagrasses, phenolic contents have been found to be low [81], with few exceptions [66]. This could explain the response of the eurysaline plants from the Chesapeake Bay, which inhabit eutrophied waters where nutrient over-enrichment is a widespread problem, but perhaps not the response of seagrass from Vulcano, where waters are oligotrophic. This needs to be rigorously tested in future experiments combining analyses of natural product contents, plant nutrition, and measures of plant productivity.

## References

[1] Lindroth R (2010). Impacts of Elevated Atmospheric CO_2_ and O_3_ on Forests: Phytochemistry, Trophic Interactions, and Ecosystem Dynamics. J. Chem. Ecol..

[2] Bidart-Bouzat MG, Imeh-Nathanie A (2008). Global change effects on plant chemical defenses against insect herbivores. J. Integr. Plant Biol..

[3] Valkama E, Korcheva J, Oksanen E (2007). Effects of elevated O_3_, alone and in combination with elevated CO_2_, on tree leaf chemistry and insect herbivore performance: a meta-analysis. Glob. Chang. Biol..

[4] Coley PD, Massa M, Lovelock CE, Winter K (2002). Effects of elevated CO_2_ on foliar chemistry of saplings of nine species of tropical tree.. Oecologia.

[5] Peňuelas J, Estiarte M (1998). Can elevated CO_2_ affect secondary metabolism and ecosystem function? Trends Ecol. Evol..

[6] Peňuelas J, Estiarte M, Llusia J (1997). Carbon-based secondary compounds at elevated CO_2_.. Photosynthetica.

[7] Stiling P, Cornelissen T (2007). How does elevated carbon dioxide (CO_2_) affect plant–herbivore interactions? A field experiment and meta-analysis of CO_2_-mediated changes on plant chemistry and herbivore performance. Glob. Change Biol..

[8] Meeham TD, Crossley MS, Lindroth RL (2010). Impacts of elevated CO_2_ and O_3_ on aspen leaf litter chemistry and earthworm and springtail productivity. Soil Biol. Biochem.. 42 (7).

[9] Bryant JP, Chapin FS, Klein DR (1983). Carbon/nutrient balance of boreal plants in relation to vertebrate herbivory.. Oikos.

[10] Mattson WJ, Julkunen-Tiitto R, Herms DA (2005). CO_2_ enrichment and carbon partitioning to phenolics: do plant responses accord better with the protein competition or the growth-differentiation balance models?. Oikos.

[11] Doney SC, Fabry VJ, Feely RA, Kleypas JA (2009). Ocean acidification: The other CO_2_ problem. *Annu. Rev. Mar. Sci.*.

[12] Royal Society (2005). Ocean acidification due to increasing atmospheric carbon dioxide..

[13] Waldbusser GG, Voigt EP, Bergschneider H, Green MA, Newell RIE (2011). Biocalcification in the Eastern Oyster (*Crassostrea virginica*) in Relation to Long-term Trends in Chesapeake Bay pH.. Estuaries and Coasts.

[14] Cai WJ, Wang Y (1998). The chemistry, fluxes, and sources of carbon dioxide in the estuarine waters of the Satilla and Althamaha Rivers, Georgia. Limnol. Oceanogr..

[15] Jiang ZJ, Huang X-P, Zhang JP (2010). Effects of CO_2_ Enrichment on Photosynthesis, Growth, and Biochemical Composition of Seagrass *Thalassia hemprichii* (Ehrenb.) Aschers. J. Integr. Plant Biol..

[16] Invers O, Zimmerman R, Alberte R, Perez M, Romero J (2001). Inorganic carbon sources for seagrass photosynthesis: an experimental evaluation of bicarbonate use in species inhabiting temperate waters. J. Exp. Mar. Biol. Ecol..

[17] Alcoverro T, Zimmerman RC, Kohrs DG, Alberte RS (1999). Resource allocation and sucrose mobilization in light-limited eelgrass *Zostera marina*. Mar. Ecol. Progr. Ser..

[18] Beer S, Rehnberg J (1997). The acquisition of inorganic carbon by the seagrass *Zostera marina*. Aq. Bot..

[19] Bjork M, Weil A, Semesi S, Beer S (1997). Photosynthetic utilisation of inorganic carbon by seagrasses from Zanzibar, East Africa. Mar. Biol..

[20] Beer S, Koch E (1996). Photosynthesis of marine macroalgae and seagrasses in globally changing CO_2_ environments. Mar. Ecol. Progr. Ser..

[21] Palacios S, Zimmerman R (2007). Response of eelgrass *Zostera marina* to CO_2_ enrichment: possible impacts of climate change and potential for remediation of coastal habitats. Mar. Ecol. Progr. Ser..

[22] Martin S, Rodolfo-Metalpa R, Ransome E, Rowley S, Buia M-C (2008). Effects of naturally acidified seawater on seagrass calcareous epibionts. Biol.. Letters,.

[23] Fabricius KE, Langdon C, Uthicke S, Humphrey C, Noonan S (2011). Losers and winners in coral reefs acclimatized to elevated carbon dioxide concentrations.. Nature Climate Change.

[24] Vizzini S, Tomasello A, Maida GD, Pirrotta M, Mazzola A (2010). Effect of explosive shallow hydrothermal vents on δ^13^C and growth performance in the seagrass *Posidonia oceanica*. J. Ecol.. 98.

[25] Valiela I, Koumjian L, Swain T, Teal JM, Hobbie JE (1979). Cinnamic acid inhibition of detritus feeding.. Nature.

[26] Harrison PG (1982). Control of microbial growth and of amphipod grazing by water-soluble com- pounds from the leaves of *Zostera marina*. Mar. Biol..

[27] Arnold T, Targett N (2002). Marine tannins: the importance of a mechanistic framework for predicting ecological roles. J. Chem. Ecol..

[28] Harrison PG, Chan AT (1980). Inhibition of the growth of micro-algae and bacteria by extracts of eelgrass (*Zostera marina*) leaves. Mar. Biol..

[29] Harrison PG, Durance C (1989). Reductions in photosynthetic carbon uptake in epiphytic diatoms by water-soluble extracts of leaves of *Zostera marina.* Mar. Biol..

[30] Ravn H, Andary C, Kovacs G, Molgaard P (1989). Caffeic acid esters as *in vivo* inhibitors of plant pathogenic bacteria and fungi. Biochem. Syst. Ecol..

[31] Vergeer LHT, Aarts TL, De Groot JD (1995). The ‘wasting disease’ and the effect of abiotic factors (light intensity, temperature, salinity) and infection with *Labyrinthula zosterae* on the phenolic content of *Zostera marina* shoots. Aq. Bot..

[32] McMillan C (1984). The condensed tannins (proanthocyanidins) in seagrasses. Aq. Bot..

[33] Quackenbush RC, Bunn D, Lingren W (1996). HPLC determination of phenolic acids in the water-soluble extract of *Zostera marina* L. (eelgrass) Aq. Bot.. 24(1).

[34] Ravn H, Pedersen MF, Borum J, Andary C, Anthoni U (1994). Seasonal variation and distribution of two phenolic compounds, rosmarinic acid and caffeic acid, in leaves and roots-rhizomes of eelgrass (*Zostera marina* L.) Ophelia.

[35] Muehlstein LK (1992). The host-pathogen interaction in the wasting disease of eelgrass, *Zostera marina.* Can. J. Bot..

[36] Den Hartog C (1996). Sudden declines of seagrass beds; “wasting disease” and other disasters.. Kirkman H (eds.).

[37] Vergeer LHT, Develi A (1997). Phenolic acids in healthy and infected leaves of *Zostera marina* and their growth limiting properties towards *Labyrinthula zosterae.* Aq. Bot..

[38] Dixon RA, Achnine L, Kota P, Liu C-J, Reddy MSS (2002). The phenylpropanoid pathway and plant defence – a genomics perspective. Mol. Plant Path..

[39] Barberi F, Innocenti F, Ferrara G, Keller J, Villari L (1974). Evolution of the Aeolian arc volcanism (Southern Tyrrhenian sea). Earth Planet Sci. Lett..

[40] Beccaluva L, Gabbianelli G, Lucchini R, Rossi PL, Savelli C (1985). Petrology and K/Ar ages of volcanic dredged from the Aeolian seamounts: implications for geodynamic evolution of the southern Tyrrhenian basin. Earth Planet Sci. Lett..

[41] Keller J (1980). The Island of Vulcano. In: L. Villari (Ed), The Aeolian Islands, an Active Volcanic Arc in the Mediterranean Sea.. Società Italiana di Mineralogia e Petrologia, Milano,.

[42] Baubron JC, Allard P, Toutain JP (1990). Diffuse volcanic emissions of carbon dioxide from Vulcano island, Italy.. Nature.

[43] Sedwick S, Stűben D (1996). Chemistry of shallow submarine warm springs in an arc-volcanic setting: Vulcano Island, Aeolian Archipelago, Italy *Marine Chemistry*.

[44] Giaccone G (1969). Associazioni algali e fenomeni secondari di vulcanismo nelle acque marine di Vulcano (Mar Tirreno).. Giornale Botanico Italiano.

[45] Kerrison P, Hall-Spencer JM, Suggestt DJ, Hepburn LJ, Steinke M (2011). Assessment of pH variability at a coastal CO_2_ vent for ocean acidification studies..

[46] Hofmann GE, Smith JE, Johnson KS, Send U, Levin LA (2011). High-Frequency Dynamics of Ocean pH: A Multi-Ecosystem Comparison.. PLoS ONE.

[47] DOE (1994). Handbook of methods for the analysis of the various parameters of the carbon dioxide system in seawater..

[48] Yao W, Byrne RH (1998). Simplified seawater alkalinity analysis: use of linear array spectrometers. Deep-Sea Res.. I 45,.

[49] Cai WJ, Wang Y (1998). The chemistry, fluxes, and sources of carbon dioxide in the estuarine waters of the Satilla and Althamaha Rivers, Georgia. Limnol. Oceanogr..

[50] Arnold TM, Tanner C, Rothen M, Bullington M (2008). Wound-induced accumulations of condensed tannins in turtlegrass, *Thalassia testudinum.* Aq. Bot..

[51] Steele L, Caldwell M, Boettcher A, Arnold T (2005). Seagrass-pathogen interactions: ‘pseudo-induction’ of turtlegrass phenolics near wasting disease lesions. Mar. Ecol. Prog. Ser..

[52] Hagerman AE, Klucher KM (1986). Tannins-protein interactions.. In: Plant Flavanoids in Biology and Medicine: Biochemical, Pharmacological and Structure-Activity Relationships; eds Cody V, Middleton E, Harborne J. Alan R Liss, New York,.

[53] Arnold TM, Appel H, Patel V, Stocum E, Kavalier A (2004). Carbohydrate translocation determines the phenolic content of *Populus* foliage: a test of the sink-source model of plant defense. New Phytol.. 164(1).

[54] Arnold TM, Tanner CE, Hatch WI (1995). Polyphenolic concentration of *Lobophora variegata* as a function of nitrogen availability. Mar. Ecol. Progr. Ser..

[55] Verges A, Becerro MA, Alcoverro T, Romero J (2007). Experimental evidence of chemical deterrence against multiple herbivores in the seagrass *Posidonia oceanica.* Mar. Ecol. Progr. Ser..

[56] Prado P, Heck KL (2011). Seagrass selection by omnivorous and herbivorous consumers: determining factors. Mar. Ecol. Progr. *Ser.*.

[57] Goecker ME, Heck KL, Valentine JF (2005). Effects of nitrogen concentrations in turtlegrass *Thalassia testudinum* on consumption by the bucktooth parrotfish *Sparisoma radians.* Mar. Ecol. Progr. Ser..

[58] Tomas F, Abbott JM, Steinberg C, Balk M, Williams SL (2011). Plant genotype and nitrogen loading influence seagrass productivity, biochemistry, and plant–herbivore interactions.. Ecology 92:9,.

[59] den Hartog C, Kuo JJ (2006). Taxonomy and Biogeography in Seagrasses in Seagrasses: Biology, Ecology and Conservation, Springer Verlag, Netherlands.

[60] Bortolus A, Iribarne OO, Martínez MM (1998). Relationship between waterfowl and the seagrass *Ruppia maritima* in a Southwestern Atlantic coastal lagoon..

[61] Idestam-Almquist J (1998). Waterfowl Herbivory on *Potamogeton pectinatus* in the Baltic Sea.. Oikos.

[62] Michot TC, Goss-Custard J, Rufino R, Luis A (1997). Carrying capacity of seagrass beds predicted for redheads wintering in Chandeleur Sound, Louisiana, USA, p. 93–102.. Effects of Habitat Loss and Change on Waterbirds. Institute of Terrestrial Ecology Symposium No. 30, Wetlands International Publication No 42 p..

[63] Michot TC, Chadwick PC (1994). Winter biomass and nutrient values of three seagrass species as potential foods for redheads (*Aythya americana* Eyton) in Chandeleur Sound, Louisiana.. Wetlands.

[64] Dorenbosch M, Bakker ES (2011). Herbivory in omnivorous fishes: effect of plant secondary metabolites and prey stoichiometry.. Freshwater Biology.

[65] Elkin RG, Rogler JC, Sullivan TW (1991). Differential response of ducks and chicks to dietary sorghum tannins.. Journal of the Science of Food and Agriculture.

[66] Cronin G, Lodge DM (2003). Effects of light and nutrient availability on the growth, allocation, carbon/nitrogen balance, phenolic chemistry, and resistance to herbivory of two freshwater macrophytes.. Oecologia 137 (1),.

[67] Ross C, Puglisi MP, Paul VJ (2008). Antifungal defenses of seagrasses from the Indian River Lagoon, Florida. Aq. Bot..

[68] Abd El-Hady HH, Daboor SM, Ghoniemy AE (2007). Nutritive and antimicrobial profiles of some seagrasses from Bardawil Lake, Egypt.. Egyptian Journal of Aquatic Research.

[69] Nicholson RL, Hammerschmidt R (1992). Phenolic Compounds and Their Role in Disease Resistance. Ann. Rev.. Phytopathology.

[70] Buchsbaum RN, Short FT, Cheney DP (1990). Phenolic-nitrogen interactions in eelgrass, *Zostera marina* L.: possible implications for disease resistance. Aq. Bot..

[71] Barbehenna RV, Constabel CP (2011). Tannins in plant–herbivore interactions.. Phytochemistry.

[72] Bennett RN, Wallsgrove RM (1994). Basic pathways for the origin of allelopathic compounds. Tansley Review No. 72. *In* Secondary Metabolites in Plant Defence Mechanisms,. Seigler DS ed.

[73] Swain T,  Rosenthal GA, Janzen DH (1979). Tannins and lignins. In Herbivores their Interaction with secondary plant metabolites..

[74] Hughes A, Williams S, Duarte C, Heck K, Waycott M (2009). Associations of concern: declining seagrasses and threatened dependent species.. Frontiers in Ecol. Environ..

[75] Valentine J, Duffy J, Larkum A, Orth R, Duarte C (2006). The central role of grazing in seagrasses ecosystems.. Seagrasses: biology ecology and conservation..

[76] Jones CG, Hartley SE (1999). A Protein Competition Model of Phenolic Allocation.. Oikos.

[77] Stamp N (2003). Out of the quagmire of plant defense hypotheses.. Q. Rev. Biol..

[78] Bazzaz FA (1997). Allocation of resources in plants: state of the science and critical questions..

[79] Herms DA, Mattson WJ (1992). The dilemma of plants – to grow or defend.. Q. Rev. Biol..

[80] Coley PD, Bryant JP, Chapin FS (1985). Resource availability and plant antiherbivore defense.. Science.

[81] Vergés A, Pérez M, Alcoverro T, Romero J (2008). Compensation and resistance to herbivory in seagrasses: induced responses to simulated consumption by fish.. Oecologia.

